# Impact of Open Proximal Contacts on Peri‐Implant Diseases: A Systematic Review and Meta‐Analysis

**DOI:** 10.1002/cre2.70278

**Published:** 2026-01-26

**Authors:** Momen A. Atieh, Maanas Shah, Abeer Hakam, Khaleifa Bohamedi, Andrew Tawse‐Smith, Nabeel H. M. Alsabeeha

**Affiliations:** ^1^ Hamdan Bin Mohammed College of Dental Medicine Mohammed Bin Rashid University of Medicine and Health Sciences Dubai UAE; ^2^ Sir John Walsh Research Institute, Faculty of Dentistry University of Otago Dunedin New Zealand; ^3^ School of Dentistry University of Jordan Amman Jordan; ^4^ College of Dentistry, Department of Restorative Dentistry Ajman University Ajman UAE

**Keywords:** dental implants, meta‐analysis, open proximal contact, peri‐implantitis, systematic review

## Abstract

**Objectives:**

The aim of this systematic review and meta‐analysis was to evaluate the impact of open proximal contacts on peri‐implant tissue changes, specifically marginal bone levels, probing pocket depth, and the incidence of peri‐implant diseases.

**Methods:**

Electronic databases were searched to identify non‐randomized observational studies comparing open and closed proximal contacts. Risk of bias was assessed using the Cochrane Collaboration's tool and data were analyzed with a statistical software.

**Results:**

Out of 276 studies initially identified, five met the inclusion criteria, involving 4882 dental implants. Meta‐analysis indicated that open proximal contacts were associated with greater, but not statistically significant, marginal bone changes (mean difference (MD) 0.07; 95% confidence interval (CI) −0.09 to 0.24; *p* = 0.38); probing pocket depths (MD 0.11; 95% CI −0.29 to 0.51; *p* = 0.59) and a higher incidence of peri‐implantitis (relative risk (RR) 1.63; 95% CI 0.88–3.02; *p* = 0.12) compared to closed contacts. Open proximal contact was associated with a significant increase in incidence of peri‐implant mucositis (RR 1.74; 95% CI 1.06–2.86; *p* = 0.03).

**Conclusions:**

Open proximal contacts are associated with increased probing pocket depths and marginal bone changes and could be a risk indicator for peri‐implant mucositis. Further research is needed to assess long‐term effects and to develop preventive measures.

## 1 Introduction

Dental implants are a well‐established and widely accepted solution for replacing missing teeth, offering high long‐term survival rates with functional and esthetic benefits (Howe et al. 2019; Atieh et al. 2009, 2010). However, a number of biological and mechanical complications may arise over time. These include peri‐implant bone loss, soft tissue inflammation, ceramic veneer chipping, screw loosening, and prosthetic component failure (Atieh et al. 2022, 2013; Pjetursson et al. 2012). Given the long‐term function expected from implant‐supported restorations, it is essential to consider the service‐related changes that may affect not only the prosthesis but also its relationship with adjacent dentition and supporting tissues. One such under‐recognized complication is the loss of proximal contact between implant restorations and adjacent teeth over time, commonly referred to as open proximal contact (Koori et al. 2010; Greenstein et al. 2016).

Open proximal contacts may develop even between contacts that were initially firm and intact, with incidence rates reported to be higher in implant restorations than those observed between natural teeth (Koori et al. 2010; Varthis et al. 2016; Jeong and Chang 2015). This phenomenon occurs more frequently on the mesial aspect of implant‐supported crowns, although distal contact loss has also been documented (Koori et al. 2010). The most widely accepted explanation is rooted in the differing biological behavior of implants and natural teeth (Richter 1989). Natural teeth exhibit physiological mesial drift as part of intra‐arch adaptation whereas osseointegrated implants remain static in the bone. This mismatch often leads to the formation of interproximal gaps adjacent to implant restorations, which can result in clinical complications such as food impaction, dental caries, soft tissue inflammation, and patient discomfort (Greenstein et al. 2016; Saber et al. 2020).

To manage this complication, several studies have proposed some restorative measures, including the use of removable retainers or splints to minimize the mesial migration of adjacent teeth and maintain tighter proximal contact at implant sites (Shi et al. 2019; Sheba et al. 2024). Such interventions, however, add financial and logistical burdens for both patients and clinicians (Abduo and Lau 2022). Importantly, while open proximal contact is classified as a prosthetic complication, its potential influence on peri‐implant soft and hard tissue health is increasingly being recognized. Some studies have reported associations between open proximal contacts and increased probing pocket depths, bleeding on probing, marginal bone loss, and other peri‐implant tissue changes (Koori et al. 2010; Saber et al. 2020; Sheba et al. 2024). Nevertheless, the evidence remains inconclusive regarding its direct relationship with clinical and radiographic peri‐implant parameters and the incidence of peri‐implant diseases. Given the growing clinical relevance and potential implications of this complication, the present systematic review aimed to critically evaluate the available evidence on the association between loss of proximal contact and peri‐implant tissue outcomes, focusing on both clinical and radiographic parameters and the development of peri‐implant diseases.

## 2 Materials and Methods

This systematic review adhered to the guidelines set forth by the Cochrane Collaboration and the Preferred Reporting Items for Systematic Reviews and Meta‐analyses (PRISMA) (Page et al. 2021). The criteria were established based on the participant, intervention, comparison, outcomes, study design framework (Higgins et al. 2024; Richardson et al. 1995), as follows:

Participant: Human adults aged ≥ 18 years with implant‐supported fixed prostheses.

Intervention: Open proximal contacts between implant‐supported fixed prostheses and natural tooth or two adjacent non‐splinted implant‐supported fixed prostheses.

Comparison: Closed proximal contacts in the same situations (i.e., between implant and tooth, or between two adjacent implants).

Outcomes: Changes in marginal bone level, probing pocket depths and incidence of peri‐implant mucositis and peri‐implantitis.

Study design: Non‐randomized observational studies.

The study was registered with the National Institute for Health Research (NIHR) under PROSPERO ID CRD420251089277. Ethical approval was not required for this systematic review.

### 2.1 Types of Studies

#### 2.1.1 Inclusion Criteria

This review included non‐randomized observational studies that compared open and closed proximal contacts between implant‐supported prostheses and either adjacent natural teeth or adjacent non‐splinted implants. This broader inclusion criterion aimed to provide a more comprehensive assessment of the clinical and biological implications of open proximal contacts on peri‐implant tissues, recognizing that both scenarios involve similar challenges in plaque control and soft tissue response. The included studies were required to report on changes in marginal bone level, probing pocket depths, or the incidence of peri‐implant diseases. No restrictions were applied regarding language or publication status.

#### 2.1.2 Exclusion Criteria

Case series, case reports, histomorphometric research, and those lacking sufficient data were excluded.

#### 2.1.3 Type of Participants

Participants were 18 years of age or older and received fixed implant‐supported restorations adjacent to either natural teeth or non‐splinted implants and in occlusion with natural or artificial tooth.

#### 2.1.4 Types of Interventions

The intervention group involved implant restorations with open proximal contacts, while implant restorations in the control group had closed proximal contacts. Implant restorations were in function for at least 1 year.

### 2.2 Outcome Measures

#### 2.2.1 Primary Outcome

Changes in marginal bone levels.

#### 2.2.2 Secondary Outcomes

Changes in probing pocket depths.

Peri‐implant mucositis rate.

Peri‐implantitis rate.

### 2.3 Search Strategy

The search protocol followed accepted practices (Faggion et al. 2013; Higgins et al. 2024). The Cochrane Central Register of Controlled Trials (CENTRAL), MEDLINE, EMBASE, and ClinicalTrials.gov were the electronic databases searched for ongoing and unpublished trials up to July 08, 2025 (Table 1). The databases were searched separately and in duplicate by two reviewers (M.A. and N.A.). Bibliographies of all eligible papers were also searched for further studies and a manual search of the last 5 years of pertinent dental journals (*Clinical Implant Dentistry and Related Research*, *Clinical Oral Implants Research*, *International Journal of Oral and Maxillofacial Implants*, *International Journal of Periodontics and Restorative Dentistry*, and *Journal of Periodontology*) was also conducted.

**Table 1:** Databases and search terms.

Databases	Keywords
Published studies PubMed (1965—July 08, 2025) EMBASE via Ovid (1947—July 08, 2025) Cochrane Central Register of Controlled Trials (CENTRAL) via Ovid (July 08, 2025)	(dental implant OR oral implant) AND (open contact OR contact loss) AND (bone changes OR peri‐implant mucositis OR peri‐implantitis) (dental adj implant OR oral adj implant).mp. AND (open adj contact OR contact adj loss).mp. AND (bone adj changes OR peri‐implant adj mucositis OR peri‐implantitis).mp. (dental adj implant OR oral adj implant).mp. AND (open adj contact OR contact adj loss).mp. AND (bone adj changes OR peri‐implant adj mucositis OR peri‐implantitis).mp.
Unpublished studies ClinicalTrials.gov (July 08, 2025)	(dental implant OR oral implant) AND (open contact OR contact loss) AND (bone changes OR peri‐implant mucositis OR peri‐implantitis)

### 2.4 Selection of Studies

The titles, abstracts, and keywords of the retrieved citations were screened separately and in duplicate by two reviewers (M.A. and N.A.). After eliminating irrelevant papers, the full texts of the remaining ones were collected. An eligibility form was used to assess potential papers for inclusion in the review. Disagreements between reviewers were resolved through discussions or by consulting a third reviewer (M.S.). When duplicate papers were selected, the one with the most adequate and relevant information was chosen. Reasons for exclusion were mentioned.

### 2.5 Data Collection

Using a data extraction form, two reviewers (M.A. and N.A.) separately gathered the following information from the included studies: (1) Study characteristics: Title, authors' names, study location, language of publication, year of publication, published or unpublished data, source of study funding, and study design. (2) Participants: Demographic characteristics, inclusion/exclusion criteria, number of participants in test and control groups, and attrition rate and reasons for dropouts. (3) Interventions: Number of implant restorations that had open proximal contact. (4) Comparison: Number of implant restorations that had closed proximal contact. (5) Outcomes: Changes in marginal bone level, probing pocket depths, and rates of peri‐implant mucositis and peri‐implantitis. (6) Length of the observation period. Any differences of opinion among reviewers were settled via consensus‐building discussions or by consulting a third reviewer (M.S.). Corresponding authors of included studies were contacted when additional information was required.

### 2.6 Quality Assessment of Included Studies

Two reviewers (M.A. and N.A.) evaluated the risk of bias for each of the included studies separately and in duplicate using the Cochrane Collaboration's Risk of Bias in non‐randomized studies of interventions version 2 (ROBINS‐I V2) tool (Higgins et al. 2024). The ROBINS‐I tool covers seven domains: (1) Confounding; (2) classification of interventions; (3) selection of participants into the study; (4) deviations from intended interventions; (5) missing outcome data; (6) measurement of outcomes; (7) selection of reported results; and overall risk of bias. Risk of bias was rated 0 for lack of information; 1 for low risk; 2 for moderate risk; 3 for serious risk, and 4 for critical risk.

### 2.7 Data Synthesis

A statistical program (Review Manager [RevMan] software, version 5.3, The Nordic Cochrane Center, The Cochrane Collaboration, Copenhagen, Denmark) was used to perform meta‐analyses for studies of similar comparisons reporting the same end measures. Continuous data, such as changes in marginal bone level, was expressed as mean difference (MD) or standardized mean difference and 95% confidence intervals (CIs). Dichotomous data such as rates of peri‐implantitis were expressed in risk ratio (RR) estimates and 95% CIs. Since study heterogeneity was anticipated, the results from multiple studies were pooled using the random‐effects model.

Because the power to detect publication bias was low (less than 10 papers), publication bias was not officially assessed (Higgins et al. 2024). The Cochran's test for heterogeneity and *I* ^2^ statistic were used to evaluate the statistical heterogeneity between various studies (Higgins et al. 2024). Significant heterogeneity was indicated by an *I* ^2^ score greater than 60. The implant served as the analysis' statistical unit. To investigate the cause of heterogeneity, the stability of the results, and the impact of the studies, a leave‐one study‐out sensitivity analysis was carried out. Sensitivity analysis was used to find out whether estimated effects changed when analyses that included studies with a high risk of bias were omitted. The GRADE criteria (risk of bias, inconsistency, imprecision, indirectness, and publication bias) were used to evaluate the certainty of evidence (Higgins et al. 2024). A software program (GRADEpro Guideline Development Tool software, McMaster University and Evidence Prime, 2021, available from gradepro.com) was used to create summary of findings table.

## 3 Results

### 3.1 Characteristics of the Study Settings

**Figure 1:**
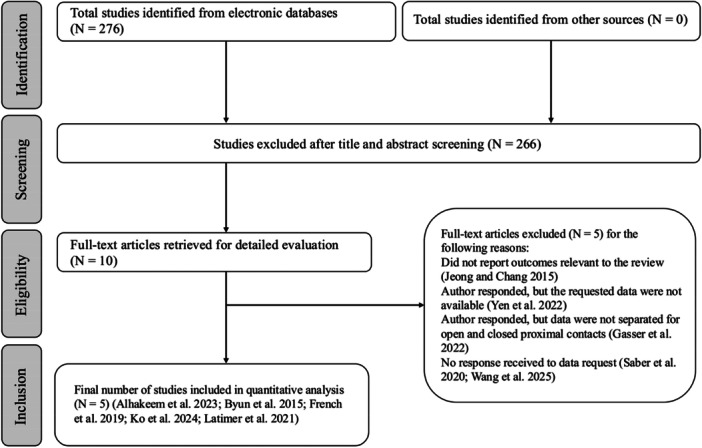
Flowchart of the search process.

A total of 276 studies were retrieved from the databases (Figure 1). After titles and abstracts were examined independently and in duplicate by two review authors (M.A. and N.A.), 10 studies were eligible for full‐text review (Alhakeem et al. 2023; Byun et al. 2015; French et al. 2019; Gasser et al. 2022; Jeong and Chang 2015; Ko et al. 2024; Latimer et al. 2021; Saber et al. 2020; Wang et al. 2025; Yen et al. 2022). One study (Jeong and Chang 2015) was excluded as it did not report on the outcomes relevant to this review. Additional information was requested from the corresponding authors of four studies (Gasser et al. 2022; Saber et al. 2020; Wang et al. 2025; Yen et al. 2022). Yen et al. (2022) responded, but the requested data were not available. Gasser et al. (2022) also replied; however, the data were not provided separately for closed and open proximal contacts. Saber et al. (2020) and Wang et al. (2025) did not respond to our email requests. Subsequently, five studies (Alhakeem et al. 2023; Byun et al. 2015; French et al. 2019; Ko et al. 2024; Latimer et al. 2021) were included in the present review (Table 2). Of the five included studies, one was conducted in Iran (Alhakeem et al. 2023), one in Korea (Byun et al. 2015), one in Canada (French et al. 2019), one in Taiwan (Ko et al. 2024), and one in the United States (Latimer et al. 2021).

**Table 2:** Characteristics of the included studies.

	Alhakeem et al. (2023)	Byun et al. (2015)	French et al. (2019)	Ko et al. (2024)	Latimer et al. (2021)
Study design	RCS	CSS	CSS	CCS	CSS
Location	Tehran University of Medical Sciences, Tehran, Iran	Chonbuk National University, Jeonju, South Korea	University of Alberta, Edmonton, Canada	Chi Mei Medical Center, Tainan, Taiwan	University of Washington, Seattle, USA
Number evaluated (participants/implants) CPC OPC	88/186 NR/164 NR/22	94/191 NR/126 NR/65	NR/4325 NR/3596 NR/729	26/39 NR/24 NR/15	61/142 15/65 46/77
Type of prostheses	Fixed dental prostheses (splinted and non‐splinted)	Fixed dental prostheses (splinted and non‐splinted) including single implant crowns	Fixed dental prostheses (splinted and non‐splinted) including single implant crowns	Single implant crowns	Fixed dental prostheses (splinted and non‐splinted) including single implant crowns
Age (years)	39.00 ± 6.00 (28–45)	56 (27–83)	NR	48.90 ± 6.95	66.60 ± 12.70
Implant location Maxilla Mandible	NR NR	91 100	2520 1805	NR NR	68 74
Implant system	a, b	NR	^a^ ^,^ ^c^	a	NR
Proximal contact position Mesial Distal	NR NR	134 57	484 66	39 0	142 142
Methods of assessment	Dental floss, manual periodontal probe	Waxed dental floss^d^, manual periodontal probe^e^, digital periapical radiograph	Satin floss (width: 0.05 mm × height: 0.004 mm)^f^, digital peri‐apical radiographs	Dental floss (70 µm)^f^, digital peri‐apical radiographs, periodontal probe	Fine, unwaxed dental floss (490 denier multi‐filament nylon), digital radiographs (vertical bitewings), periodontal probe (UNC 15 color‐coded probe)
Changes in MBL (mm) CPC OPC	NR NR	0.66 ± 0.88 0.88 ± 1.11	0.40 ± 0.65 0.43 ± 0.74	0.13 ± 0.41 0.02 ± 0.39	0.80 ± 1.30 1.30 ± 1.80
Implant ‐ PPD (mm) CPC OPC	NR NR	3.37 ± 0.99 3.37 ± 1.23	NR NR	2.60 ± 0.93 2.40 ± 0.51	3.60 ± 1.00 4.10 ± 1.30
Peri‐implant mucositis rate N (%) CPC OPC	NR NR	NR NR	NR NR	NR NR	16 (24.6) 33 (42.8)
Peri‐implantitis rate N (%) CPC OPC	15 (9.1) 3 (13.6)	NR NR	NR NR	NR NR	9 (13.9) 18 (23.4)
Follow‐up period (months)	84	57	54	24	60

Abbreviations: CCS, case control study; CPC, closed proximal contact; CSS, cross sectional study; MBL, marginal bone level; NR, not reported; OPC, open proximal contact; PPD, probing pocket depth; RCS, retrospective cohort study.

^a^ Straumann implant system, Institut Straumann AG, Basel, Switzerland.

^b^ Dentium implant system, Dentium Co. Ltd., Seoul, South Korea.

^c^ Nobel Biocare implant system, Nobel Biocare AB, Gothenburg, Sweden.

^d^ Dental floss, Oksen Preden Co., Uiwang, South Korea.

^e^ Hu‐Friedy Mfg. Co. LLC, Chicago, IL, USA.

^f^ Oral‐B, Procter & Gamble, Boston, MA, USA.

Four studies (Alhakeem et al. 2023; Byun et al. 2015; Ko et al. 2024; Latimer et al. 2021) were conducted in university settings, while one study (French et al. 2019) involved implants placed in private practice. All studies employed a parallel‐group design. Three of the included studies (Alhakeem et al. 2023; Byun et al. 2015; Latimer et al. 2021) were supported by internal university grants, whereas the remaining two (French et al. 2019; Ko et al. 2024) did not report any funding information.

### 3.2 Characteristics of Participants at Baseline

Inclusion criteria

1. Aged ≥ 18 (Ko et al. 2024), ≥ 21 (Latimer et al. 2021), or ≤ 40 years (Alhakeem et al. 2023).
2. Patients must have at least one dental implant that has been functionally loaded with a final restoration for a minimum of 1 year. The implant must be adjacent to either at least one natural tooth on one or both sides (Alhakeem et al. 2023; Byun et al. 2015; French et al. 2019; Ko et al. 2024; Latimer et al. 2021) or a non‐splinted implant (Alhakeem et al. 2023) and must be in functional occlusion with either a natural or prosthetic tooth in the opposing arch.
3. Good oral hygiene, defined as full‐mouth plaque and bleeding scores below 25%, along with adherence to routine dental check‐ups (Ko et al. 2024).
4. Patients have a minimum of 20 natural teeth present at the time of implant placement (Alhakeem et al. 2023).

Exclusion criteria

1. Uncontrolled diabetes (HbA1c > 7%) (Alhakeem et al. 2023; Latimer et al. 2021).
2. Osteoporosis (Alhakeem et al. 2023; Latimer et al. 2021).
3. Systemic diseases or medications affecting bone remodeling and mucosal healing (e.g. non‐steroidal anti‐inflammatory drugs, bisphosphonates, and/or chronic steroids) (Alhakeem et al. 2023; Ko et al. 2024; Latimer et al. 2021).
4. Patient who had received antibiotic therapy within 2 months prior to the examination (Alhakeem et al. 2023; Ko et al. 2024).
5. History of radiotherapy and chemotherapy (Ko et al. 2024).
6. Current smokers (Ko et al. 2024) or former smokers with less than 3 months of cessation prior to implant placement (Latimer et al. 2021).
7. Parafunctional activities (Ko et al. 2024).
8. Current pregnancy (Alhakeem et al. 2023; Ko et al. 2024; Latimer et al. 2021) or breastfeeding (Alhakeem et al. 2023).
9. Implants adjacent to teeth with dental caries or plaque‐retentive dental restorations, as well as implants that had undergone any surgical intervention following placement (Alhakeem et al. 2023).

### 3.3 Characteristics of the Interventions

Patients who met the inclusion criteria were invited to attend a follow‐up examination and some participants were already attending routine recall visits at intervals of 3–12 months (Byun et al. 2015; Latimer et al. 2021). Implants had been placed following manufacturer protocols (Byun et al. 2015; French et al. 2019; Ko et al. 2024) and implant‐supported prostheses were delivered after a healing period of 3–6 months (Byun et al. 2015; Ko et al. 2024). Baseline information, including patient demographics, implant type, number of implants placed, and any history of ridge augmentation, was obtained from clinical records (Alhakeem et al. 2023; French et al. 2019). Clinical and radiographic measurements were collected by trained and calibrated examiners following a standardized protocol (Alhakeem et al. 2023; Latimer et al. 2021). In one study (Byun et al. 2015), assessments were performed by calibrated examiners who were not involved in the initial treatment. Inter‐examiner calibration was reported in one study (Latimer et al. 2021). In contrast, another study (French et al. 2019) involved a single examiner who both placed the implants and conducted the clinical and radiographic assessments, with no intra‐examiner calibration reported.

Proximal contact status was assessed using dental floss, and categorized as closed or “tight” if resistance was felt during flossing or open if no resistance was encountered (Alhakeem et al. 2023; Byun et al. 2015; French et al. 2019; Ko et al. 2024; Latimer et al. 2021). Different types of dental floss were used across studies, including waxed floss (Oskan Preden, Uiwang, Korea) (Byun et al. 2015), satin floss (Oral‐B, 0.05 mm width) (French et al. 2019), 70 µm floss (Oral‐B, Boston, MA, USA) (Ko et al. 2024), and fine unwaxed multi‐filament nylon floss (490 denier) (Latimer et al. 2021). The prevalence of open proximal contacts at the implant level ranged from 11.8% after an 8‐year follow‐up (Alhakeem et al. 2023) to 54.2% after more than 10 years of functional loading (Latimer et al. 2021). An increasing trend in proximal contact loss was observed over time (French et al. 2019; Ko et al. 2024; Latimer et al. 2021), with approximately 50% of contacts lost by 9 years after prosthesis delivery (Byun et al. 2015). Open contacts were more frequently observed on the mesial surfaces than distal ones (Byun et al. 2015; French et al. 2019; Latimer et al. 2021), in the mandible compared to the maxilla (20% vs. 15%) (French et al. 2019), and in anterior than posterior implants (18% vs. 14%) (French et al. 2019). In addition, higher rates of open contacts were reported in platform‐switched and single implants compared to platform‐matched and splinted implants (Latimer et al. 2021).

Radiographic evaluation of marginal bone level changes was performed using vertical bitewings (Latimer et al. 2021) or digital radiographs with the parallel technique (Byun et al. 2015; French et al. 2019; Ko et al. 2024). Linear measurements were taken from digitized images using image analysis software (Byun et al. 2015; French et al. 2019; Latimer et al. 2021). Marginal bone levels were measured from implant platform or abutment junction to the first bone to implant contact (Byun et al. 2015; French et al. 2019; Ko et al. 2024; Latimer et al. 2021), with adjustments made for tissue‐level implant collar length where applicable (French et al. 2019). Probing pocket depths were measured at six sites per implant (mesiofacial, mid‐facial, distofacial, mesiolingual, and distolingual) using light probing force (0.25 N) (Ko et al. 2024). Two studies (Byun et al. 2015; Ko et al. 2024) reported no significant association between proximal contact status and peri‐implant tissue condition, including probing pocket depth and bleeding on probing. In contrast, Latimer and coworkers (2021) found that loss of proximal contact was associated with increased probing depths and marginal bone loss.

Diagnosis of peri‐implant diseases was based on probing pocket depths and radiographic marginal bone levels according to consensus definitions (Alhakeem et al. 2023) and the 2017 World Workshop on Periodontal and Peri‐implant Diseases (Latimer et al. 2021). While the presence of one or more open proximal contacts was significantly associated with peri‐implant diseases, it was not linked to implant loss. No implant failures were observed during either the 2‐year follow‐up (Ko et al. 2024) or the 6–8 year follow‐up period (Alhakeem et al. 2023).

Regarding patient‐reported outcomes, Latimer et al. (2021) reported that 32.6% of patients were aware of open contacts and 56.5% experienced food impaction. Similarly, Byun et al. (2015) found food impaction in 47% of all proximal embrasures, with a significantly higher prevalence in the open contact group compared to sites with intact contacts (63% vs. 39%).

### 3.4 Outcome Measures

#### 3.4.1 Primary Outcome

Changes in marginal bone level.

#### 3.4.2 Secondary Outcomes

Changes in probing pocket depth.

Peri‐implant mucositis rate.

Peri‐implantitis rate.

### 3.5 Characteristics of Outcome Measures

#### 3.5.1 Primary Outcome Measure

- Marginal bone level was measured in millimeters using vertical bitewings (Latimer et al. 2021), and digital radiographs with a parallel film holder (Byun et al. 2015; French et al. 2019; Ko et al. 2024). Measurements were taken mesially and distally as the vertical distance from the implant platform to the most apical point of bone‐to‐implant contact (French et al. 2019; Latimer et al. 2021). Calibration for radiographic magnification was performed using the known implant length and diameter (Byun et al. 2015; French et al. 2019; Ko et al. 2024).

#### 3.5.2 Secondary Outcome Measures

- Probing pocket depths were measured in millimeters using a University of North Carolina‐15 color‐coded probe (Latimer et al. 2021), Williams probe (Hu‐Friedy, Chicago, IL) (Alhakeem et al. 2023) and a calibrated PGF/W periodontal probe with 1 mm markings (Hu‐Friedy, Chicago, IL) (Byun et al. 2015).
- Rates of peri‐implant mucositis and peri‐implantitis were reported in two studies (Alhakeem et al. 2023; Latimer et al. 2021). The definitions for both conditions followed the criteria established by the 2017 World Workshop on the Classification of Periodontal and Peri‐implant Diseases and Conditions (Berglundh et al. 2018; Renvert et al. 2018). Peri‐implant mucositis was defined as the presence of clinical signs of inflammation without bone loss beyond initial remodeling. Peri‐implantitis was defined by the presence of clinical signs of inflammation, increased probing pocket depth, and progressive bone loss. In cases where baseline radiographs or periodontal charting were not available, peri‐implantitis was diagnosed based on either: Radiographic bone loss ≥ 3 mm, probing pocket depth ≥ 6 mm and bleeding on probing, or radiographic bone loss ≥ 3 mm, probing pocket depth ≥ 4 mm, and bleeding and/or suppuration on probing (Sanz et al. 2018). Peri‐implant health was defined by the absence of clinical signs of inflammation, no bleeding on probing or suppuration, and no bone loss beyond initial remodeling (i.e., ≤ 2.0 mm) (Renvert et al. 2018).

### 3.6 Risk of Bias in Non‐Randomized Studies

**Figure 2:**
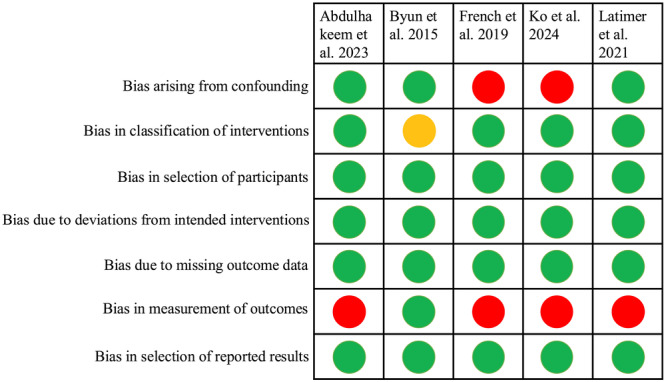
Assessment of risk of bias of the non‐randomized studies presented with low (green), moderate (orange), and serious (red) risk of bias.

All the included studies were non‐randomized observational studies. Overall, four studies (Alhakeem et al. 2023; French et al. 2019; Ko et al. 2024; Latimer et al. 2021) were judged to be at serious risk of bias, while the remaining one (Byun et al. 2015) was graded at moderate risk of bias concerns (Figure 2).

Bias due to confounding: Two studies (French et al. 2019; Ko et al. 2024) were judged to have a serious risk of bias as they did not attempt to control for confounding variables. In contrast, three other studies (Alhakeem et al. 2023; Byun et al. 2015; Latimer et al. 2021) made efforts to adjust for potential confounders such as age, gender, implant location, arch, platform type, retention system, and loading time, and were therefore rated as having a low risk of bias.

Bias in classification of intervention: The interventions were clearly described in all studies except one (Byun et al. 2015), where the exact time of proximal contact loss was not reported. As result, this study was assessed as having a moderate risk of bias in this domain, while the remaining studies (Alhakeem et al. 2023; French et al. 2019; Ko et al. 2024; Latimer et al. 2021) were judged to have a low risk of bias.

Bias in selection of participants: All eligible participants with dental implants who met the inclusion criteria were enrolled in the studies. Therefore, all studies were judged to have a low risk of bias in this domain.

Bias in outcome measurement: Only one study (Byun et al. 2015) reported blinding of outcome assessors and was therefore rated as having a low risk of bias. The remaining studies (Alhakeem et al. 2023; French et al. 2019; Ko et al. 2024; Latimer et al. 2021) did not report assessor blinding and were judged to be at serious risk of bias in this domain.

Bias due to deviations from intended interventions, missing outcome data or selective reporting: None of the included studies showed deviations from the intended interventions, high attrition rates, or evidence of selective outcome reporting. Therefore, all were assessed as having a low risk of bias in these domains.

### 3.7 Sample Size Calculation

Sample size calculation was reported in two studies (Alhakeem et al. 2023; Latimer et al. 2021), while it was not addressed in the other three studies (Byun et al. 2015; French et al. 2019; Ko et al. 2024).

### 3.8 Effects of Interventions

The current review included a total of 4883 implants, of which 1050 exhibited open proximal contacts (Table 3). All results were analyzed and reported at the implant level.

**Table 3:** Summary of findings: Open proximal contact versus closed proximal contact adjacent to implant restorations.

Outcome	Number of studies	Relative effect (95% CI)	Anticipated absolute effects^a^ (95% CI)		Certainty of the evidence (Grade)^b^
			CPC	OPC	
Changes in marginal bone levels (mm)	4 studies	Not estimable	The mean ranged across control groups from 0.04 to 0.66	MD 0.07 higher (0.09 lower to 0.24 higher)	⊕⊝⊝⊝ Very low^c^, ^d^, ^e^
Changes in probing pocket depths (mm)	3 studies	Not estimable	The mean ranged across control groups from 2.6 to 3.6	MD 0.11 higher (0.29 lower to 0.51 higher)	⊕⊝⊝⊝ Very low^c^, ^d^, ^e^
Peri‐implant mucositis	1 study	RR 1.74 (1.06–2.86)	246 per 1000	182 more per 1000 (15 to 458 more)	⊕⊕⊝⊝ Low^d^, ^f^
Peri‐implantitis	2 studies	RR 1.63 (0.88–3.02)	92 per 1000	58 more per 1000 (11 fewer to 186 more)	⊕⊕⊝⊝ Low^d^, ^g^
GRADE Working Group grades of evidence					
**High certainty:** We are very confident that the true effect lies close to that of the estimate of the effect.					
**Moderate certainty:** We are moderately confident in the effect estimate: the true effect is likely to be close to the estimate of the effect, but there is a possibility that it is substantially different.					
**Low certainty:** Our confidence in the effect estimate is limited: the true effect may be substantially different from the estimate of the effect.					
**Very low certainty:** We have very little confidence in the effect estimate: the true effect is likely to be substantially different from the estimate of effect.					

Abbreviations: CI, confidence interval; CPC, closed proximal contact; MD, mean difference; OPC, open proximal contact; RR, risk ratio.

a The risk in the intervention group (and its 95% CI) is based on the assumed risk in the comparison group and the relative effect of the intervention (and its 95% CI).

^b^ None of the studies suffered from indirectness or detected publication bias.

^c^ Downgraded one level due to risk of bias: Inadequate adjustment for confounding.

^d^ Downgraded one level due to risk of bias: Outcome assessor was not blinded.

^e^ Downgraded one level due to inconsistency: Heterogeneity was detected.

^f^ Downgraded one level due to imprecision: The effect estimate is based on a single study.

^g^ Downgraded one level due to inconsistency: Only two studies contributed to the analysis, limiting assessment of heterogeneity.

#### 3.8.1 Changes in Marginal Bone Levels

**Figure 3:**
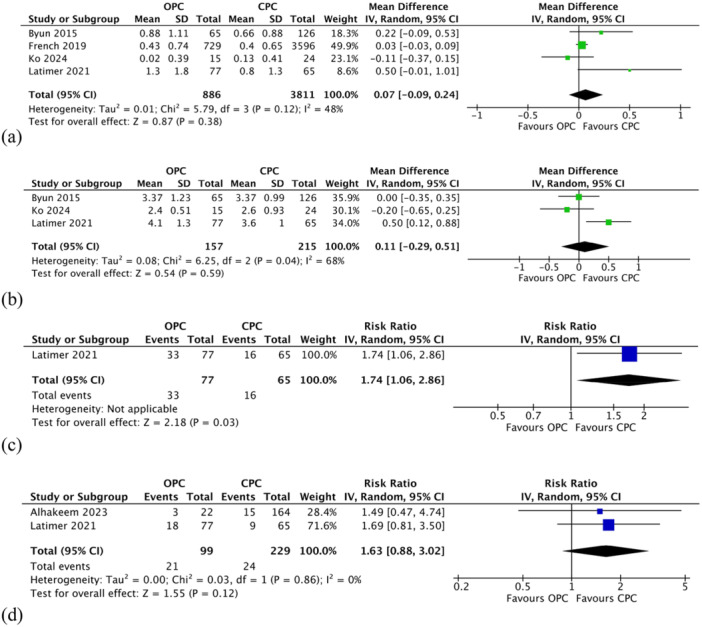
Comparison: Open proximal contact versus closed proximal contact. Primary outcome: (a) Changes in marginal bone levels. Secondary outcomes: (b) Changes in probing pocket depths (c) peri‐implant mucositis rate (d) peri‐implantitis rate. τ: Kendall tau; CI, confidence interval; CPC, closed proximal contact; IV, inverse variance; OPC, open proximal contact; SE, standard error; z, z test.

Marginal bone levels were reported in four studies (Byun et al. 2015; French et al. 2019; Ko et al. 2024; Latimer et al. 2021). The meta‐analysis showed that implants with open proximal contacts had greater changes in marginal bone levels compared to those with closed contacts. However, the difference was not statistically significant (MD 0.07; 95% CI −0.09 to 0.24; *p* = 0.38; Figure 3a). Moderate heterogeneity was detected across the studies (*χ* ^2^ = 5.79, df = 3 [*p* = 0.12]; *I* ^2^ = 48%).

#### 3.8.2 Changes in Probing Pocket Depths

Three studies (Byun et al. 2015; Ko et al. 2024; Latimer et al. 2021) reported on changes in probing pocket depths. The overall meta‐analysis indicated that implants with open proximal contacts exhibited greater changes in probing pocket depths compared to those with closed contacts; however, this difference was not statistically significant (MD 0.11; 95% CI −0.29 to 0.51; *p* = 0.59; Figure 3b). Substantial heterogeneity was observed across the studies (*χ* ^2^ = 6.25, df = 2 [*p* = 0.04]; *I* ^2^ = 68%).

#### 3.8.3 Peri‐Implant Mucositis and Peri‐Implantitis Rates

Peri‐implant mucositis was reported in one study (Latimer et al. 2021). Among implants with open proximal contact, 33 cases of peri‐implant mucositis were observed compared to 16 cases in the closed contact group. The difference was statistically significant (RR 1.74; 95% CI 1.06–2.86; *p* = 0.03; Figure 3c). Peri‐implantitis rates were reported in two studies (Alhakeem et al. 2023; Latimer et al. 2021). Although the incidence was higher in the open contact group, the difference between the groups was not statistically significant (RR 1.63; 95% CI 0.88–3.02; *p* = 0.12; Figure 3d). There was no evidence of heterogeneity (*χ* ^2^ = 0.03, df = 1 (*p* = 0.86); *I* ^2^ = 0%).

#### 3.8.4 Sensitivity Analyses

The leave‐one‐out sensitivity analysis indicated that the overall effect size for changes in marginal bone levels remained stable throughout the sensitivity checks. The pooled MD ranged from 0.03 to 0.16, and heterogeneity (*I* ^2^) varied between 23% and 63% depending on which study was excluded. Excluding any single study did not substantially alter the overall results or statistical significance. None of the included studies was identified as an outlier or had a disproportionate impact on the overall estimate. The difference in marginal bone levels between the open and closed contact groups remained statistically non‐significant regardless of which study was removed (Table 4).

**Table 4:** Leave‐one study‐out sensitivity analysis: Marginal bone levels.

Removed study	Overall MD (95% CI)	*p* value	Heterogeneity
Byun et al. (2015) French et al. (2019) Ko et al. (2024) Latimer et al. (2021)	0.04 (−0.15, 0.24) 0.15 (−0.18, 0.48) 0.16 (−0.08, 0.39) 0.03 (−0.08, 0.14)	*p* = 0.66 *p* = 0.36 *p* = 0.19 *p* = 0.59	*p* = 0.11; *I* ^2^ = 54% *p* = 0.06; *I* ^2^ = 63% *p* = 0.10; *I* ^2^ = 56% *p* = 0.28; *I* ^2^ = 23%

Abbreviations: CI, confidence interval; MD, mean difference.

## 4 Discussion

### 4.1 Summary of Main Results

This systematic review compared outcomes of implant restorations with open versus closed proximal contacts over time. The outcomes assessed included changes in marginal bone levels, probing pocket depths, and the incidence of peri‐implant mucositis and peri‐implantitis. Implants with open proximal contacts showed greater changes in marginal bone levels and probing pocket depths compared to those with closed contacts; however, these differences were not statistically significant. Open proximal contacts were also associated with higher rates of both peri‐implant mucositis and peri‐implantitis. The increase in peri‐implant mucositis was statistically significant, whereas the increase in peri‐implantitis was not.

### 4.2 Quality of Evidence

In this systematic review, we included cross‐sectional, prospective, and retrospective cohort studies that met predefined stringent selection criteria, aimed at enhancing the methodological quality and minimizing potential sources of heterogeneity. Despite these efforts, the limited number of studies included in the meta‐analysis on peri‐implant diseases precluded a robust assessment of heterogeneity; an expected limitation given the variability in diagnostic criteria and outcome definitions of peri‐implant diseases across studies. Moderate to substantial heterogeneity was observed in several of the other analyses, suggesting that differences in study design, patient populations, follow‐up durations or outcome measurement methods may have contributed to variability in the results. Despite the limited number of eligible studies and variations in study methodologies, the clinical and radiographic outcomes were reported in a sufficiently comparable manner to permit a cautious meta‐analysis, and the pooled estimates should be interpreted in light of these constraints.

To minimize potential confounding, most of the included studies enrolled participants who were under regular oral health maintenance and exhibited good oral hygiene, typically defined by plaque and bleeding scores below 25%. Given that poor oral hygiene is a well‐established risk factor for peri‐implant diseases (Derks et al. 2016; Atieh et al. 2022, 2013), the inclusion of patients with adequate oral hygiene allowed a more focused evaluation of the impact of open proximal contacts on clinical and radiographic parameters, including peri‐implant diseases incidence. Moreover, this review included one study (Alhakeem et al. 2023) that evaluated open proximal contacts between implant‐supported prostheses adjacent to natural teeth and between two adjacent non‐splinted implants. Although both contact types were included due to their shared clinical relevance, particularly the similar challenges they pose in plaque control and tissue response, their biological and clinical implications may not be entirely identical. This broader inclusion likely introduced some heterogeneity and should be considered a limitation. Future research that clearly distinguishes between theses contact types in study design and reporting would help clarify any differential effects on peri‐implant tissue outcomes.

Four studies (Alhakeem et al. 2023; French et al. 2019; Ko et al. 2024; Latimer et al. 2021) were judged to have a serious risk of bias, primarily due to issues related to confounding and outcome measurement. However, it is noteworthy that the two studies reporting on peri‐implantitis rates (Alhakeem et al. 2023; Latimer et al. 2021) employed statistical adjustments for potential confounding variables, which may lend greater certainty to the evidence. Overall, the certainty of evidence was rated as low to very low, primarily due to methodological limitations such as risk of bias in outcome assessment, lack of assessor blinding, small number of studies, and the moderate to substantial heterogeneity observed across analyses. Consequently, the findings of this review should be interpreted with caution.

### 4.3 Applicability of Evidence

While various prosthetic designs and features have been extensively studied for their influence on soft and hard tissue changes and consequently on the development of peri‐implant diseases (Atieh et al. 2023; Janda and Mattheos 2024), fewer studies have specifically investigated the association between open proximal contacts and changes in clinical and radiographic peri‐implant parameters. Several narrative and systematic reviews (Abduo and Lau 2022; Bento et al. 2023; Ghasemi et al. 2022; Greenstein et al. 2016; Manicone et al. 2022; Pappous et al. 2024; Sheba et al. 2024; Varthis et al. 2019) have addressed open proximal contacts as a well‐recognized prosthetic complication in implant dentistry. However, the primary focus of these studies was on the prevalence rather than on the clinical outcomes such as peri‐implant inflammation and marginal bone loss. Open proximal contacts are known to negatively affect periodontal health (Hancock et al. 1980; Jernberg et al. 1983), but their potential impact on peri‐implant tissues has only gained attention in recent years (Greenstein et al. 2016). As such, the clinical significance of this prosthetic complication in relation to peri‐implant health remains an emerging area of interest.

The reported prevalence of open proximal contacts in implant restorations ranged from 34% to 66% (Varthis et al. 2019). A recent systematic review by Sheba et al. (2024) found that the likelihood of contact loss was 2.1 times higher at the mesial surface of restorations compared to the distal surface, consistent with the prevalence trends observed in most of the included studies. A lower prevalence rate of 17%, however, was reported in the study of French et al. (2019) with 4325 implants, which could be related to the relatively shorter follow‐up period of 4 years. Several factors have been associated with the overtime development of open proximal contacts, including long‐term occlusal changes, flaring of adjacent teeth, craniofacial and jaw growth, and mesial drift of natural teeth (Heij et al. 2006; Saber et al. 2020; Sheba et al. 2024; Wei et al. 2008). A consistent finding across these studies was the strong positive correlation between longer follow‐up durations and increased rates of proximal contact loss (Byun et al. 2015; French et al. 2019; Ko et al. 2024; Sheba et al. 2024). To mitigate the risk of proximal contact loss over time, preventive strategies such as splinting of adjacent teeth, the use of occlusal guards or Essix retainers have been suggested to limit tooth migration (Jeong and Chang 2015; Kandathilparambil et al. 2020; Sheba et al. 2024).

Patient awareness of open proximal contacts is a notable finding, particularly in relation to dis‐satisfaction with food impaction. The incidence of food impaction is reported to occur 2.2 times more frequently in sites with open contacts compared to those with closed contacts (Byun et al. 2015). Although food impaction may lead to patient dissatisfaction and discomfort, the included studies in this review did not demonstrate a statistically significant association between food impaction and peri‐implant health parameters. These findings are consistent with previous investigations, which also failed to establish conclusive evidence linking food impaction to adverse peri‐implant outcomes (Chanthasan et al. 2022; Jeong and Chang 2015; Varthis et al. 2016).

This review demonstrated a trend toward greater marginal bone loss and increased probing pocket depths in sites with open proximal contacts; however, these differences did not reach statistical significance when compared to sites with closed contacts. These findings contrast with previous studies (Koori et al. 2010; Saber et al. 2020), which reported significantly deeper probing depth, increased clinical attachment loss, higher plaque and gingival indices and greater marginal bone loss in association with open contacts. It is likely that the inclusion of patients with well‐maintained oral hygiene and regular supportive periodontal care could have mitigated the potential negative effects of open proximal contacts on peri‐implant tissue health. These findings, certainly, underscore the critical role of oral hygiene and maintenance protocols in the long‐term health and success of dental implants.

Moreover, this review identified open proximal contacts between implant restorations and adjacent teeth as a risk indicator for peri‐implant mucositis, demonstrated with significant increase in bleeding scores and deeper probing pocket depths. These findings highlight the importance of maintaining optimal plaque control and attending regular follow‐up in preserving peri‐implant tissue health and early detection of proximal contact loss. In this context, the use of screw‐retained implant restorations may be the preferred retention method, as they facilitate easier retrieval and timely correction of open proximal contacts.

The present review has several limitations that should be acknowledged. First, the inclusion of cross‐sectional studies limits the ability to establish causality between open proximal contacts and peri‐implant diseases, as these designs are inherently suited to assessing associations. Second, the inclusion of retrospective studies introduces potential biases related to incomplete or missing data and the lack of examiner calibration. Additionally, methodological heterogeneity, particularly with respect to the varying techniques used to assess proximal contacts, might have influenced the consistency of findings across studies. Furthermore, some participants had a history of treated periodontitis, a known risk factor for increased susceptibility to peri‐implant diseases (Atieh et al. 2022, 2013), which could have introduced confounding effects. Nonetheless, all participants were reported to have received regular supportive periodontal care, thereby allowing a more controlled assessment of the influence of proximal open contacts on peri‐implant tissue health, independent of other major contributing factors.

## 5 Conclusions

Within the limitations of this systematic review, open proximal contacts are associated with increased probing pocket depths and marginal bone changes and could be a potential risk indicator for peri‐implant mucositis but the evidence regarding the overall peri‐implant tissue health remains inconclusive. Further well‐designed longitudinal studies with larger cohorts are still needed to better assess the impact of proximal open contacts on peri‐implant health and to develop effective preventive measures.

## Author Contributions

**Momen A. Atieh:** concept/design, data collection, data analysis/interpretation, drafting article, critical revision of article, approval of article. **Maanas Shah:** data analysis/interpretation, critical revision of article, approval of article. **Abeer Hakam:** critical revision of article, approval of article. **Khaleifa Bohamedi:** data collection, data analysis/interpretation, critical revision of article, approval of article. **Andrew Tawse‐Smith:** critical revision of article, approval of article. **Nabeel H. M. Alsabeeha:** critical revision of article, approval of article.

## Funding

The authors received no specific funding for this work.

## Ethics Statement

The authors have nothing to report.

## Consent

The authors have nothing to report.

## Conflicts of Interest

The authors declare no conflicts of interest.

## Data Availability

The data that support the findings of this study are available on the request from the corresponding author. The data are not publicly available due to privacy or ethical approval.
